# Advancing and Translating Knowledge in Vascular Medicine

**DOI:** 10.3389/fcvm.2014.00006

**Published:** 2014-10-29

**Authors:** Marc Husmann, Matthias Barton

**Affiliations:** ^1^Clinic of Angiology, University Hospital Zürich, Zürich, Switzerland; ^2^Molecular Internal Medicine, University of Zürich, Zürich, Switzerland

**Keywords:** endovascular therapy, Andreas Grüntzig, intraluminal therapy, atherosclerosis, coronary artery disease, translational medicine, angiology, vascular medicine

## Abstract

For centuries, physicians have depended on the use of written information to gain knowledge. Book printing and binding introduced by Gutenberg in the fifteenth century revolutionized and accelerated the distribution of information. Advancing medical knowledge and progress is not only linked to the scientific quality of a discovery determining it will be accepted by the peers but also by its communication and sharing of new findings with the medical community. All these factors determine whether new knowledge will advance and improve clinical practice, medical education, and ultimately, patient care, and human health. In the past decade medical publishing has witnessed a revolution with regard to the instant, online availability of published “open access” information, which can be accessed and printed from any computer connected to the internet. As an example, how language and availability of printed information may affect distribution of knowledge, we discuss the publication of the first results of balloon angioplasty in patients with peripheral vascular disease 40 years ago by Andreas Grüntzig, M.D. at the University of Zürich. *Vascular Medicine*, as part of *Frontiers in Cardiovascular Medicine*, will provide open access provided to all published content for sharing and distributing new and most up-to-date information on clinical practice and medical knowledge in vascular medicine. We anticipate that the ongoing transformation of scientific publishing through open access will further accelerate this process and make new knowledge available even faster. Immediate, unrestricted, and rapid access to the most current knowledge published will play a role in maintaining and advancing human vascular health across the globe.

## Introduction: Communication in Medicine

For centuries, physicians have depended on the use of written information to gain knowledge and the first known examples were written on clay or papyrus. Book printing and binding – introduced by Gutenberg in the 15th century – revolutionized and accelerated the distribution of information. The first medical journals were launched already in the eighteenth century (1–4) and by the middle of the nineteenth century “*if you were a scientist you were expected to read French, English, and German*” (5). At the time, the German language was considered the primary language of international scientific communication because German scientists held the leading position in many disciplines, including chemistry, mathematics, and physics (6). In the nineteenth century and early twentieth century, German was also the language of scientific publication in Vienna, then the reputed center of medicine, psychology, and psychiatry, as well as in Czechoslovakia, Poland, Scandinavia, and the Netherlands (6). Even in English-speaking countries such as the UK and the United States, scientists were expected to read German to keep up with the developments in their fields (6, 7). After the Nazi regime came into power in 1933, German successively lost its predominance as a scientific language, scientists – who had left Germany and took exile in the United States or the UK – started publishing their work in English (6, 8). However, not until 1960 English fully took over as the primary language of science (9). In fact, many German medical journals with a long-publishing tradition successively changed to publish in English to increase the global visibility of their articles published (9). Today, more than 98% of all scientific articles published are published in English (8).

## Communicating Progress in Medicine

Communication of scientific discoveries and medical progress is directly linked to population health. In that context, translational medicine is concerned with the translation of research discoveries into clinical application for the prevention, diagnosis, and treatment of human diseases. On February 12, 1974, when at the University of Zürich German physician Andreas Grüntzig, M.D. (1939–1985, Figure 1), for the first time applied a balloon-tipped dilating catheter to treat a patient with peripheral vascular disease (10, 11), he could not know that 40 years later his method would be considered as one of the most important therapeutic advances and outstanding examples of translational medicine of the twentieth century (12). Though was a major breakthrough for vascular medicine, and Grüntzig and his colleague, chemist Heinrich Hopff (with whom he had developed the new polyvinyl chloride (PVC) balloon device (10)), wanted to publish both the balloon catheter concept and the first patient cases treated with this new method. This was at a time when the first Xerox machines had arrived in offices just a few years before, when there were no computers, calculators, or an internet; at a time when statistical analysis had to be done without the help of statistics computer programs, when scientific manuscripts and their carbon copies had be written on a typewriter, and when figures had to be hand-drawn with rulers and black ink.

**Figure 1:**
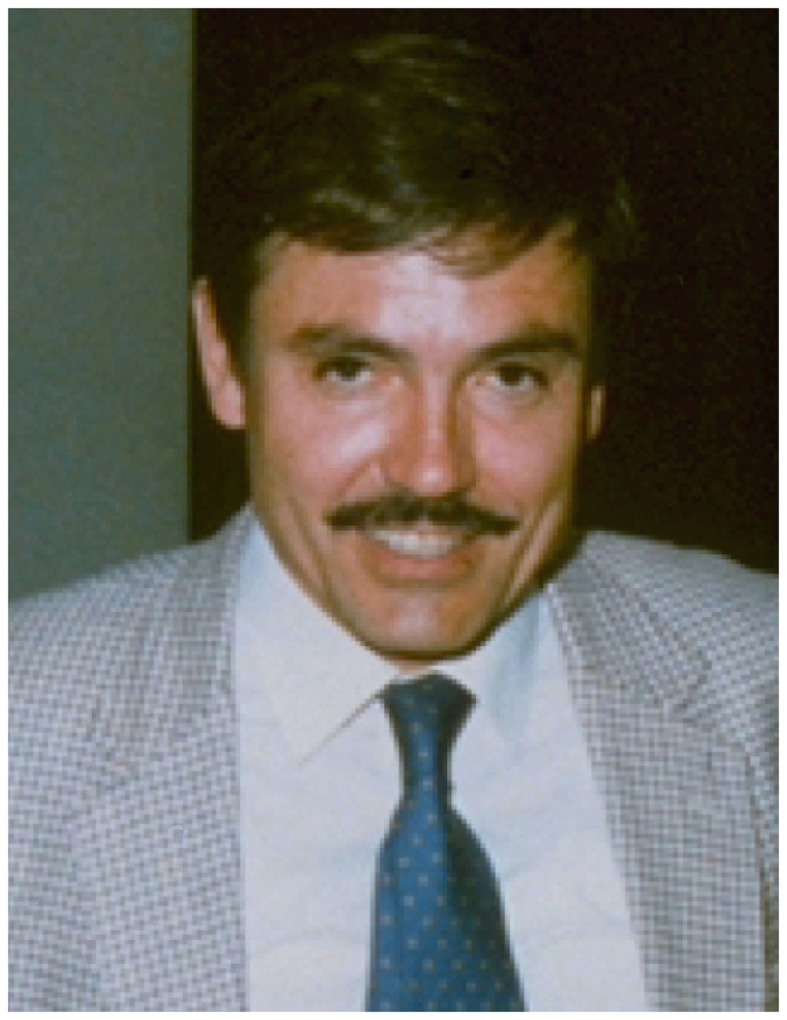
**Andreas Grüntzig, M.D. (1939–1985), photograph taken 1982 in Nürnberg, Germany**. Photograph courtesy of Felix Mahler, M.D., and used with his permission.

**Figure 2:**
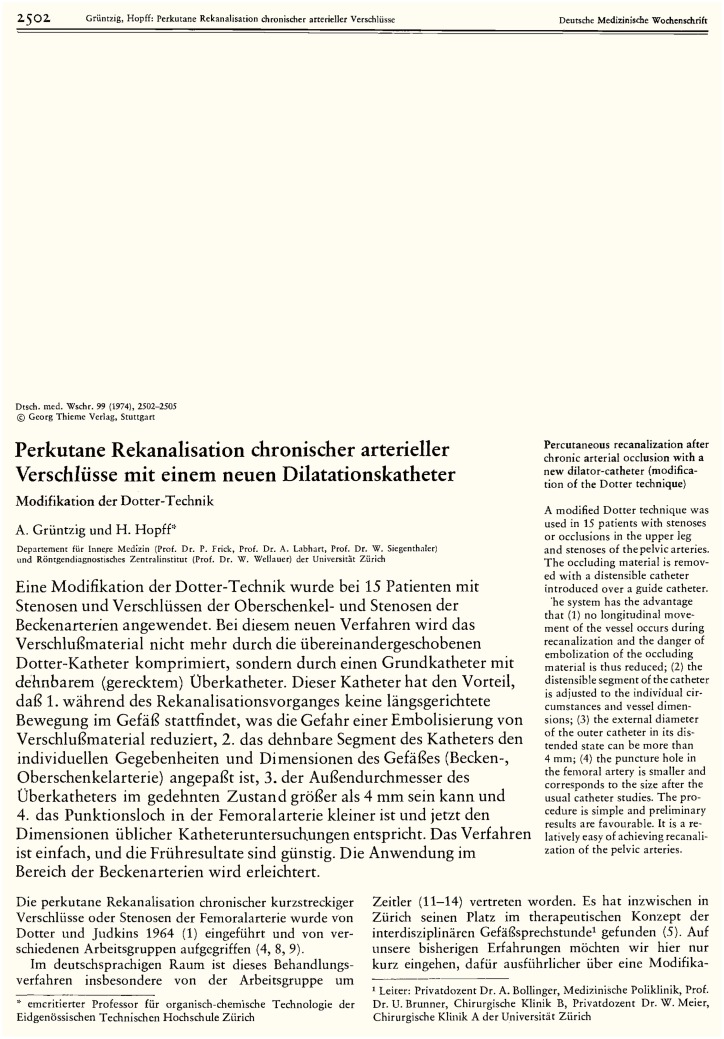
**First page of the original article published in 1974 in German by Andreas Grüntzig, M.D. and Heinrich Hopff, Ph.D., reporting Grüntzig’s the new method of balloon angioplasty and results of the first 15 patients with peripheral artery disease successfully treated with this new therapy**. Note the insert of an English abstract on the right of the page. Reproduced from Grüntzig and Hopff (10), with permission of the publisher.

For the publication of their findings Grüntzig and Hopff chose a journal that only published articles in German (10). Automatically, this meant that access to this new medical information was going to be neither rapid nor global. In fact, access to the new information was delayed by the printing process which in those days could require several months up to 2 years. The latter happened to one of the research groups who independently discovered the vascular activity of an endothelium-derived peptide (13), which was later identified as endothelin (14). Moreover, the publication by Grüntzig and Hopff could be read only by those individuals proficient in the German language, and was restricted to German-speaking countries in which printed journal copies were distributed among its subscribers. Fortunately, the article did contain an English abstract (Figure 2). In October of 1977, Grüntzig went global with his intellectual property by filing a patent application for the balloon angioplasty device in Switzerland, Germany, France, the United Kingdom, the United States, and Japan (15). However, this patent was not issued until 1980, 6 years after Grüntzig had begun treating patients using his new method. Regardless of the patent being issued, it took a full 5 years until Grüntzig’s new balloon angioplasty method finally reached the United States (16). The restriction of dissemination of Grüntzig’s new treatment of peripheral vascular disease within the medical community can serve as a good example of how access to important new medical information can be delayed namely by limiting the communication required for the attention an information may deserve. Ironically, the journal originally chosen by Grüntzig and Hopff in 1974 to publish his first balloon angioplasty cases even today still publishes most of its articles in German; however, it may occasionally consider manuscripts written in English.

## Open Access to Knowledge in Vascular Medicine

Advancing medical knowledge and progress is not only linked to the scientific quality of a discovery, which will determine its acceptance by the peers, but also by its communication and sharing of new findings with the medical community. All these factors determine whether new knowledge will be able to advance and improve clinical practice, medical education, and ultimately, patient care and human health. Medical publishing has recently witnessed a revolution through open access publishing and the online availability of published information, which can be accessed and printed instantaneously from any computer connected to the internet. The advantages of such a development have been realized more than a decade ago (17) and are obvious: articles published “open access” allow physicians to retrieve the most current information instantaneously without delay, utilize said information for clinical decision making, teaching of medical students and fellows. Finally, physicians and medical scientists can use open access journals to communicate their own research or to share clinical knowledge with their colleagues.

Printed medical journals and text books by nature have a limited number of printed pages. This limitation, which is simply due to economic reasons and costs, often prevents important and noteworthy articles to be published in widely read journals. In fact, due to the printed pages limit many journals in medicine or science can accept only 20% or less of all manuscripts submitted. As a result, many otherwise fine articles are published with considerable delay or sometimes cannot even be published at all in a particular journal. Unfortunately, this also slows down advancement of medical progress and understanding of new scientific questions being addressed in a manuscript. Some scientists in the field have even abandoned submitting manuscripts to some of the key journals, as they feel that the way by which these journals handle manuscript submissions distorts the scientific process (18, 19).

## A New Era for Vascular Medicine

The key element of quality control in medical and scientific publishing always has been and will remain high-level, independent peer-review of studies by experts knowledgeable in a particular field, regardless whether a manuscript contains data or content related to medicine or science. In most of today’s medical journals, authors do not learn about the identity of those who review or reviewed a particular manuscript. Sometimes, they can be direct competitors, working in the same area, which may go unnoticed by the Editors. In addition, reviewers normally report their manuscript assessments to the editors who will then make a decision about the fate of the manuscript which will then be communicated to the corresponding author. While there is no need to criticize this approach, it often does lead to a considerable delay until a final decision can be reached, particularly, when several revisions of a manuscript are required. What is and will remain important, though is the independence and the high scientific expertise of any editor or reviewer assessing a manuscript submitted for review.

As with all Frontiers journals, we are taking the same new approach with *Vascular Medicine* to increase transparency of the review and decision processes and to shorten the review process while maintaining a high quality of the scientific peer-review. Implementing the Frontiers interactive review, authors are required to directly interact with reviewers and the Editor after the manuscript has been submitted. This approach not only dramatically shortens the time required to address issues raised by the reviewers but also allows authors to directly communicate with their reviewers who at that stage remain anonymous. Only after the paper has been accepted for publication authors will learn the identity of their reviewers, and names and affiliations will be listed on the published manuscript.

## A New Journal: Goals and Expectations

We anticipate and hope that *Vascular Medicine* will develop into an open access knowledge platform with a strong clinical focus allowing physicians, health professionals, and vascular disease specialists to obtain and communicate information relevant to teaching and practicing vascular medicine. Readers and authors will have access to information on atherosclerotic vascular disease affecting the aorta and peripheral, renovisceral, and extracranial arteries, as well as related clinical complications such as acute and chronic critical limb ischemia and arterial aneurysms. Moreover, the journal offers articles on medical and interventional treatment of vascular diseases, particularly, studies on endovascular surgery and vascular interventions (20). In addition to atherosclerotic vascular disease, one of the main areas in vascular medicine, the Journal also covers other kinds of vascular diseases involving the macro- or microcirculation, vascular malformations, venous disease, and lymphatic diseases (21). The Journal will also feature articles on primary and secondary vascular disease prevention (22–24), new therapies (25–27), translational medicine (28), and medical education in the field of vascular medicine.

Although *Vascular Medicine* is primarily focusing on clinical medicine, human trials, and translational clinical research (12, 29), manuscripts reporting results from preclinical and experimental studies will be considered as long they are related to vascular disease and advance the understanding of the pathophysiology of the related disease condition or help to develop new treatments (28). Articles made available in *Vascular Medicine* are meant to continuously increase insights in the understanding of disease and treatment and to foster medical education – all to advance clinical practice and patient care in vascular medicine.

**Figure 3:**
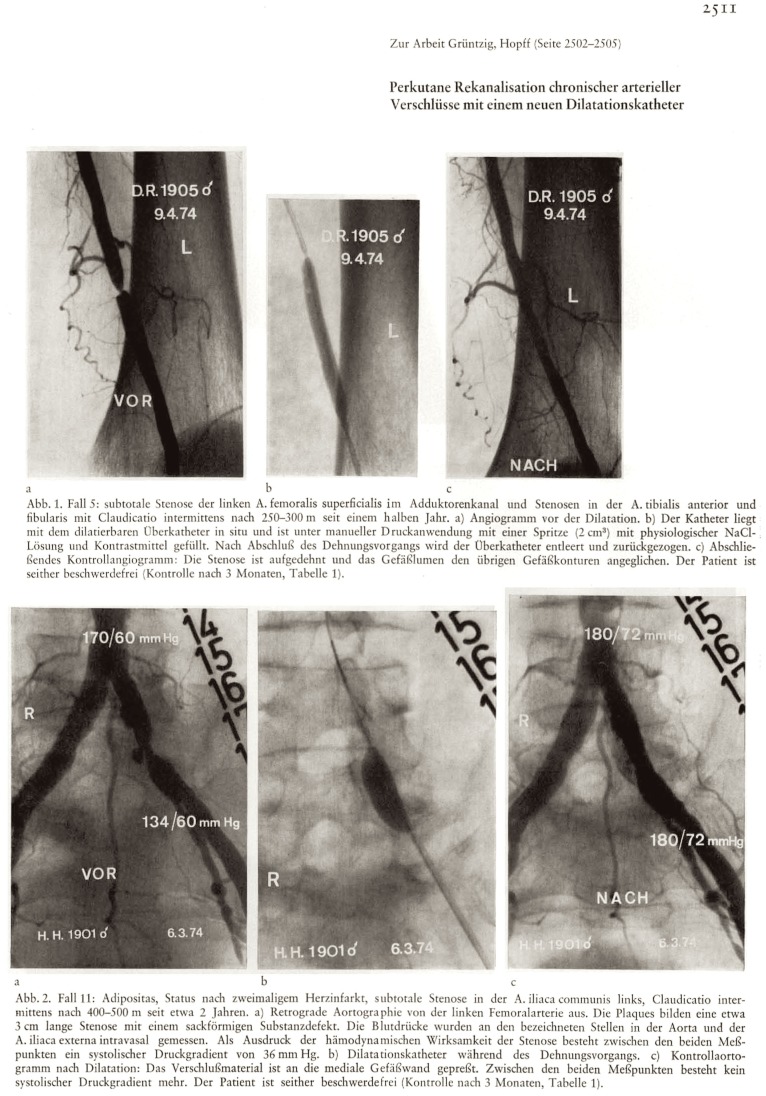
**Supplemental information (“Zur Arbeit Grüntzig, Hopff”) to the article depicted in Figure 2, showing arterial angiograms of two patients with peripheral artery disease before (left), during (middle), and after (right) percutaneous balloon angioplasty performed by Andreas Grüntzig, M.D**. The first angiogram in panel 1 (Abb. 1) indicates left superficial femoral artery stenosis in a 71-year-old man, the procedure was performed on April 9, 1974. The left angiogram panel 2 (Abb. 2) indicates a high-grade stenosis of the left common iliac artery in a 73-year-old male patient, the procedure was performed on March 6, 1974. Note the differently sized angioplasty balloons, both in diameter and length. The German figure legend indicates that both patients were free of symptoms on follow-up 3 months after the procedure. Reproduced from Grüntzig and Hopff (10), with permission of the publisher.

Forty years ago the vascular medicine pioneer Andreas Grüntzig, M.D., at our institution published the first results of balloon angioplasty treatment of patients with peripheral vascular disease (Figure 3) in a journal that was only available in German-speaking countries and to readers proficient in the German language (10). Times have changed. As the Editors of *Vascular Medicine*, we are confident that open access provided to its content published in today’s global language of science (8) will advance communication and distribution of new and most up-to-date information on clinical practice and medical knowledge in vascular medicine (17, 30). We anticipate that the ongoing transformation of scientific publishing through open access – which *Frontiers in Cardiovascular Medicine* has become part of – will further accelerate this process to make new information available even faster. Immediate, unrestricted, and free global access to the most current published knowledge will play a role in maintaining and advancing human vascular health.

## Financial Disclosure and Competing Interests Statement

Dr. Husmann has relationships with drug and device companies including Abbott Endovascular, Medtronic, EV3, Cordis, Sanofi Aventis, Daiichi Sankyo, Bayer, Boeringer Ingelheim, and AstraZeneca. In addition to being investigator involved in endovascular and drug trials involving these companies relationships include consultancy service, research grants, lecture honoraria, and travel fees and membership in advisory boards. Dr. Barton has no competing interests or relationships to disclose.

## References

[1] Warren J Remarks on angina pectoris. N Engl J Med Surg (1812) 1:1–11. 10.1056/NEJM181201010010101 14005036

[2] Anonymous. The Lancet. Sunday, October 5, 1823 – politics: enlightened liverymen! Lancet (1823) 1:10–12. Available from: http://www.sciencedirect.com/science/article/pii/S0140673601188380

[3] Kohane IS Drazen JM Campion EW A glimpse of the next 100 years in medicine. N Engl J Med (2012) 367:2538–9. 10.1056/NEJMe1213371 23268669

[4] Sarkowski H The growth and decline of German scientific publishing 1850-1945. In: Fredriksson EH, editor. A Century of Science Publishing. IOS Press (2001). 25 p. Available from: http://ebooks.iospress.nl/Download/Pdf/29499

[5] Gordin MD Scientific Babel: How Science was Done Before and After Global English. 1st ed Chicago: Chicago of University Press (2015). p. 1–424. Available from: http://www.press.uchicago.edu/ucp/books/book/chicago/S/bo14504917.html

[6] Elsevier Publishers. A short history of Elsevier. Celebration of the 125th Anniversary of Elsevier and the 425th Anniversary of the House of Elsevier. Amsterdam: Elsevier Publishers (2005). p. 1–12. Available from: http://www.elsevier.com/__data/assets/pdf_file/0014/102632/historyofelsevier.pdf

[7] Müller DC Duff MC Stern KL Timeline: 200 years of the New England Journal of Medicine. N Engl J Med (2012) 366:e3 10.1056/NEJMp1114819 22216863

[8] Engber D How did English get to be the international language of science? Everyone got mad at the Germans. Popular Sci (2013). Available from: http://www.popsci.com/article/science/fyi-how-did-english-get-be-international-language-science

[9] Baethge C The languages of medicine. Dtsch Arztebl Int (2008) 105:37–40. 19633751 10.3238/arztebl.2008.0037 PMC2696676

[10] Grüntzig A Hopff H Perkutane Rekanalisation chronischer arterieller Verschlüsse mit einem neuen Dilatationskatheter. Modifikation der Dotter-Technik. Dtsch med Wschr (1974) 99:2502–5. 10.1055/s-0028-1108161 4434847

[11] Grüntzig J Der Pioneer der kardialen Ballondilatation: Andreas Grüntzig, mein Bruder. Kardiologieforum (2008) 2:54–63.

[12] King SB Translational research: then and now. J Am Coll Cardiol Intv (2009) 2:1165–6. 10.1016/j.jcin.2009.09.004 19926065

[13] O’Brien RF Robbins RJ McMurtry IF. Endothelial cells in culture produce a vasoconstrictor substance. J Cell Physiol (1987) 121:263–70. 10.1002/jcp.1041320210 3114270

[14] Yanagisawa M Kurihara H Kimura S Tomobe Y Kobayashi M Mitsui Y A novel potent vasoconstrictor peptide produced by vascular endothelial cells. Nature (1988) 332:411–5. 10.1038/332411a0 2451132

[15] Grüntzig A Gleichner H Katheteranordnung zum Oeffnen und Verschliessen von Hohlräumen. Patentschrift Nr. 616 337 (issued 31 March 1980), Schweizerische Eidgenossenschaft, Bundesamt für Geistiges Eigentum. Bern (1980). Available from: http://worldwide.espacenet.com/publicationDetails/originalDocument?FT=D&date=19800331&DB=EPODOC&locale=en_EP&CC=CH&NR=616337A5&KC=A5&ND=4

[16] Monagan D Williams DO Journey into the Heart: A Tale of Pioneering Doctors and Their Race to Transform Cardiovascular Medicine. New York, NY: Gotham Books (2007). p. 1–325.

[17] Edejer TT Disseminating health information in developing countries: the role of the internet. BMJ (2000) 321:797–800. 10.1136/bmj.321.7264.797 11009519 PMC1118616

[18] Sample I Nobel winner declares boycott of top science journals: Randy Schekman says his lab will no longer send papers to nature, cell and science as they distort scientific process (9 December). The Guardian (2013). Available from: http://www.theguardian.com/science/2013/dec/09/nobel-winner-boycott-science-journals

[19] Schekman R How journals like nature, cell and science are damaging science: the incentives offered by top journals distort science, just as big bonuses distort banking (9 December). The Guardian (2013). Available from: http://www.theguardian.com/commentisfree/2013/dec/09/how-journals-nature-science-cell-damage-science

[20] Husmann MJ Barton M Jacomella V Silvestro A Amann-Vesti BR. Long-term effects of endovascular angioplasty on orthostatic vasocutaneous autoregulation in patients with peripheral atherosclerosis. J Vasc Surg (2006) 44:993–7. 10.1016/j.jvs.2006.06.038 17098532

[21] Husmann MJ Barton M Amann-Vesti BR Franzeck UK. Postural effects on interstitial fluid pressure in humans. J Vasc Res (2006) 43:321–6. 10.1159/000093197 16682804

[22] Barton M Husmann MJ Effects of obesity on mortality in patients with unstable angina or non-ST-elevation myocardial infarction. Eur Heart J (2007) 28:2950 10.1093/eurheartj/ehm460 17959620

[23] Husmann M Keller M Barton M Artériopathies athérosclérotiques et monoxyde d’azote (NO): l’importance clinique d’une espérance de vie plus longue et l’obésité. Forum Med Suisse (2007) 7:1008–11.

[24] Barton M. Prevention and endothelial therapy of coronary artery disease. Curr Opin Pharmacol (2013) 13:226–41. 10.1016/j.coph.2013.05.005 23742924

[25] Husmann M Barton M. Therapeutical potential of direct thrombin inhibitors for atherosclerotic vascular disease. Expert Opin Investig Drugs (2007) 16:563–7. 10.1517/13543784.16.5.563 17461731

[26] Jacomella V Corti N Husmann M. Novel anticoagulants in the therapy of peripheral arterial and coronary artery disease. Curr Opin Pharmacol (2013) 13:294–300. 10.1016/j.coph.2012.12.005 23333175

[27] Malmström RE Godman BB Diogene E Baumgärtel C Bennie M Bishop I Dabigatran – a case history demonstrating the need for comprehensive approaches to optimize the use of new drugs. Front Pharmacol (2013) 4:39. 10.3389/fphar.2013.00039 23717279 PMC3653065

[28] Bolon B Altrock B Barthold SW Baumgarth N Besselsen D Boivin G Advancing translational research. Science (2011) 33:1516–7. 10.1126/science.331.6024.1516-b 21436422

[29] Berro M Burnett BK Fromell GJ Hartman KA Rubinstein EP Schuff KG The clinical and translational science award experience. Acad Med (2011) 86:217–23. 10.1097/ACM.0b013e3182045059 21169787 PMC13220793

[30] Afarikumah E. Electronic health in Ghana: current status and future prospects. Online J Public Health Inform (2014) 5:230. 10.5210/ojphi.v5i3.4943 24678382 PMC3959911

